# Accounting for unmet needs resulting from cancer-related cognitive impairment

**DOI:** 10.1007/s11764-025-01769-6

**Published:** 2025-03-11

**Authors:** Darren Haywood, Alexandre Chan, Raymond J. Chan, Evan Dauer, Haryana M. Dhillon, Ashley M. Henneghan, Maryam B. Lustberg, Moira O’Connor, Janette L. Vardy, Susan L. Rossell, Nicolas H. Hart

**Affiliations:** 1 Human Performance Research Centre, INSIGHT Research Institute, Faculty of Health, University of Technology Sydney (UTS), Moore Park, Sydney, NSW 2030 Australia; 2 Department of Mental Health, St Vincent’s Hospital Melbourne, Fitzroy, VIC Australia; 3 Department of Psychiatry, Faculty of Medicine, Dentistry and Health Sciences, University of Melbourne, Fitzroy, VIC Australia; 4 School of Population Health, Faculty of Health Sciences, Curtin University, Bentley, WA Australia; 5 School of Pharmacy and Pharmaceutical Sciences, University of California, Irvine, CA USA; 6 Caring Futures Institute, College of Nursing and Health Sciences, Flinders University, Adelaide, SA Australia; 7 Psycho-Oncology Cooperative Research Group, Faculty of Science, School of Psychology, University of Sydney, Sydney, Australia; 8 School of Nursing, University of Texas at Austin, Austin, TX USA; 9 Department of Oncology, Dell Medical School, The University of Texas at Austin, Austin, TX USA; 10 Yale School of Medicine, New Haven, CT USA; 11 Faculty of Medicine and Health, The University of Sydney, Sydney, Australia; 12 Centre for Mental Health and Brain Sciences, Swinburne University of Technology, Hawthorn, VIC Australia; 13 Cancer and Palliative Care Outcomes Centre, Faculty of Health, Queensland University of Technology (QUT), Brisbane, QLD Australia; 14 Exercise Medicine Research Institute, School of Medical and Health Science, Edith Cowan University, Joondalup, WA Australia; 15 Institute for Health Research, University of Notre Dame Australia, Fremantle, WA Australia

**Keywords:** Cancer-related cognitive impairment, Needs, Unmet needs, Assessment, Cancer

## Abstract

**Purpose:**

Cancer-related cognitive impairment (CRCI) causes a wide range of unmet needs for cancer survivors. It is unknown which clinical, demographic, cognitive, and psychological factors underpin and account for these various unmet needs. This study aimed to (*a*) identify factors *associated* with CRCI-related unmet needs, and (*b*) establish the most pertinent factors that *account* for CRCI-related unmet needs.

**Methods:**

Four hundred and fifty-six (*n* = 456) cancer survivors responded to a range of demographic and clinical questions, as well as measures of CRCI-related unmet needs (MASCC COG-IMPACT), perceived cognitive impairment (PROMIS-COG), and psychological distress (DASS-21). Descriptive statistics, bivariate correlations, and feed-forward multiple regression analyses were completed.

**Results:**

Cognitive impairment severity (*r* = 0.39 to 0.59; *p* < 0.01), psychological distress (*r* = 0.36 to 0.58; *p* < 0.01), and time since diagnosis (*r* =  − 0.11 to − 0.20; *p* < 0.05 to *p* = 0.02) were significantly associated with CRCI-related unmet needs across all domains. Age (*r* =  − 0.10 to − 0.22; *p* < 0.001 to *p* = 0.03), stage of cancer at initial diagnosis (*r* = 0.10 to 0.13; *p* < 0.001 to *p* = 0.04), stage of cancer at most progressed (*r* = 0.11 to 0.18; *p* < 0.001 to *p* = 0.03), and sex (*r* = 0.12; *p* = 0.01; females experiencing greater unmet needs than males), were significantly associated with one or more domains of unmet need. Cognitive impairment severity and psychological distress were the most pertinent factors accounting for CRCI-related unmet needs (*R*^*2*^ = 0.245, *F*_(3, 487)_ = 48.96, *p* < 0.001 to *R*^*2*^ = 0.474, *F*_(3, 487)_ = 114.81, *p* < 0.001), explaining 24.5% to 47.4% of the variance.

**Conclusion:**

Cognitive impairment severity and psychological distress were the most key factors in accounting for CRCI-related unmet needs. Other variables, while associated with CRCI-related unmet needs, did not provide additional predictive utility.

**Implications for Cancer Survivors:**

The results may inform the choice of supportive care targets, and future strategies, to improve supportive care for people experiencing cancer-related cognitive impairment.

## Introduction

Cancer-related cognitive impairment (CRCI) refers to cancer-related changes in memory, executive functioning, speed of information processing, decision-making, and other cognitive functioning that is experienced by up to 75% of cancer survivors [1–4]. CRCI is theorised to be caused and maintained by the physiological effects of cancer and cancer treatments as well as the severity of psychological distress (i.e. depression, anxiety, and stress), and the impairments can last decades after treatment completion [1–3, 5, 6]. CRCI can be measured and inferred via objective neurocognitive assessment as well as through subjective self-report methods, which are often only weakly correlated, and subjective and objectively determined CRCI may differ in their aetiology [1–3]. However, both objective and subjectively inferred CRCI can have considerable negative consequences on the lives of cancer survivors, with quantitative and qualitative evidence consistently showing CRCI to commonly impact activities of daily living, social and relational functioning, occupational and vocational functioning, and psychological well-being [3, 7–11]. Due to the impacts of CRCI, cancer survivors experiencing CRCI often have a range of unmet supportive needs resulting from their cognitive changes [8, 9, 12].

Unmet supportive care needs are common among cancer survivors with or without CRCI [9, 13, 14]. However, cancer survivors with CRCI can experience unique unmet supportive care needs that are directly driven by their cognitive changes and the impacts these changes have on their lives and overall well-being [3, 8, 9, 12]. For example, cancer survivors with CRCI may experience significant supportive care needs relating to their return or performance at work, or related to their difficulties participating in social interactions due to verbal fluency challenges [9, 15].

CRCI-related unmet supportive care needs may be highly dependent on a cancer survivor's clinical and personal context [9]. For example, a cancer survivor with CRCI currently in employment may experience greater, or different, unmet supportive care needs when compared to a cancer survivor with CRCI who is retired. Relatedly, the severity of CRCI may impact the degree and type of unmet supportive care needs a cancer survivor may have [16]. Further, demographic characteristics such as age and sex are commonly associated with the severity and presence of general unmet needs [17, 18], and may partly account for the unmet needs resulting from CRCI. For example, older age is commonly associated with lesser social engagement [19], and thus older cancer survivors with CRCI may experience fewer CRCI-related unmet needs in domains such as verbal communication. Clinical characteristics, such as time since diagnosis, are also commonly associated with general unmet needs within cancer survivors [20–22] and may potentially also partly account for CRCI-related unmet needs through mechanisms such as having additional time to develop and utilise CRCI impact mitigation strategies. Distress is also common in cancer survivorship [3, 23, 24] and may also contribute to the development, exacerbation, and perpetuation of CRCI-related unmet needs, beyond its impact on cognitive impairment severity, through mechanisms such as negative self-perception and reduced self-efficacy [3, 25, 26].

To date, there has been no available purpose-built measure of CRCI-related unmet needs available, and thus understanding the clinical, demographic, cognitive, and psychological factors associated with CRCI-related unmet supportive care needs was unable to be explored. Recently, a purpose-built unmet needs assessment for CRCI impact has been developed and validated; the Multinational Association of Supportive Care in Cancer (MASCC)—Unmet Needs Assessment of Cancer-Related Cognitive Impairment Impact (MASCC COG-IMPACT) [16]. The MASCC COG-IMPACT allows for the assessment of key factors associated with CRCI-related unmet supportive care needs across multiple domains [16].

Understanding the key factors that account for CRCI-related unmet needs, including and beyond cognitive impairment severity, is critical to inform practitioners of who might be at significant risk of experiencing CRCI-related unmet needs, and what factors are important to consider or prioritise when developing and providing assessment, referral, and supportive care practices to cancer survivors experiencing CRCI.

The aims of this study were (*a*) to identify clinical, demographic, cognitive and psychological factors *associated* with CRCI-related unmet needs, and (*b*) to establish the most pertinent clinical, demographic, cognitive, and psychological variables that *account* for CRCI-related unmet needs.

## Methods

### Design

A cross-sectional self-report study design was used. Ethics approval was obtained from St. Vincent’s Hospital Melbourne Human Research Ethics Committee prior to data collection (PID05582), and participants provided informed consent prior to participating in the research.

### Participants

Participants were 18 years or older, had a prior diagnosis of cancer (any type), had completed curative intent cancer treatment, had no current evidence of disease, personally identified as experiencing CRCI, and were fluent in English (reading and speaking). The only exclusion criterion was the self-reported diagnosis of another neurocognitive or neurological disorder.

Participants who self-identified as experiencing CRCI were recruited, as opposed to only those who met pre-defined neuropsychological ‘cut-off’ scores due to the qualitatively reported significance of the lived-experience of perceived cancer-related cognitive *changes* (i.e. before versus after a cancer diagnosis) on unmet supportive care needs*.* Participants self-identified as currently experiencing memory, decision-making, verbal fluency, processing speed, and/or reasoning impairments following their cancer treatment completion.

### Recruitment

Participants were recruited via Prolific [27], a highly respected research participant sourcing platform. Prolific has been constantly shown to be valid and reliable, and the platform has been extensively used for neuropsychological research, including within oncology [16, 28–31].

### Measures

A subset of an existing dataset from the development and validation of the MASCC COG-IMPACT was used for this study [16]. The self-report survey included a range of demographic and clinical questions (e.g. age, sex, level of education, employment status, days since initial diagnosis, cancer stage at initial diagnosis, cancer stage at most progressed), the MASCC COG-IMPACT [16], the Patient-Reported Outcomes Measurement Information System-Cognitive Function Scale 8a (PROMIS-COG) [32], and the Depression, Anxiety and Stress Scale (DASS-21) [33].

The MASCC COG-IMPACT is a 55-item, eight subscale (Finding Meaning and Enjoyment in Activities, Relational Difficulties, Occupational/Vocational Functioning, Psychological Challenges, Verbal Communication Challenges, Social Functioning and Withdrawal, and Informational Needs), validated self-report measure of CRCI-related unmet needs [16]. A CRCI-related unmet needs score can be calculated for each MASCC COG-IMPACT subscale, and an overall CRCI-related unmet needs score can be calculated across all subscales (see [34]). PROMIS-COG [32] is a highly validated self-report assessment of cognitive function for cancer survivors recommended by the Neuroscience Initiative Working Group to be used for CRCI research [35, 36]. The PROMIS-COG provides an overall score across its items. DASS-21 is a highly validated and utilised measure of psychological distress, including three subscales, Depression, Anxiety, and Stress [33] Subscale scores as well as an overall score can be calculated using the DASS-21 [33].

### Analysis

Descriptive statistics and frequency analyses were used to describe the sample, and correlational analyses were used in this study to achieve the study’s aims. Specifically, means, standard deviations, counts, ranges, and percentages are used to detail the sample’s characteristics, Pearson’s bivariate correlation analyses (for continuous variables and ordinal variables) and point-biserial correlation analyses (for binary variables) were used to identify demographic and clinical characteristics associated with CRCI-related unmet needs as appropriate, and nine feed-forward multiple regression analyses (one per the MASCC COG-IMPACT subscale and one for the overall MASCC COG-IMPACT score) were used to establish the most pertinent variables that can account for CRCI-related unmet needs. The strength of effect for the bivariate correlations was interpreted as follows; small *r* < 0.3, medium *r* = 0.31 to *r* = 0.69, large *r* ≤ 0.70 [37].

Feed-forward multiple regression analyses were chosen as it was best suited to both (a) establish the most pertinent factors associated with CRCI-related unmet needs, and (b) mitigate potential multicollinearity issues related to correlated predictor variables by not forcing each predictor to be included as per a standard multiple regression analysis. For the feed-forward multiple regression analyses, the criterion variables were the MASCC COG-IMPACT subscales, and overall unmet needs score, and the predictor variables were the age, sex, level of education, employment status (yes/no), days since diagnosis, cancer stage at diagnosis, cancer stage at most progressed, the PROMIS-COG, and the DASS-21 subscales. Predictors were maintained in the final models if they contributed to accounting for a significant amount of unique variance in the criterion. A Bonferroni-corrected [38] alpha value for multiple analyses (nine feed-forward multiple regression analyses) of 0.006 was used for the assessment of each overall final model, with a standard 0.05 alpha level used for the assessment of the unique predictive utility of each predictor variable, and for the bivariate correlation analyses.

## Results

### Participants

A total sample of 456 cancer survivors were used for this study, out of the 491 in the full dataset [16], as 35 participants did not provide complete data across all variables required for this research. Sample characteristics are reported in Table 1. Participants were from 23 different countries and represented 18 different primary cancer types.

**Table 1:** Sample characteristics

Characteristic	Mean (SD)/count
Age (years)	*M* = 44.8 years (SD = 14.8)
Sex at birth	
Male	118 (25.9%)
Female	338 (74.1%)
Ethnicity	
Caucasian	275 (60.3%)
African/African American	134 (29.4%)
Asian	17 (3.7%)
Hispanic or Latino	12 (2.6%)
Native American/American Indian	3 (0.7%)
Other	15 (3.3%)
Primary cancer type	
Breast	153 (33.6%)
Prostate	20 (4.4%)
Bowel/colorectal	28 (6.1%)
Melanoma	17 (3.7%)
Lung	23 (5.0%)
Lymphoma	35 (7.7%)
Leukaemia	26 (5.7%)
Brain	11 (2.4%)
Pancreatic	3 (0.7%)
Myeloma	3 (0.7%)
Cervical	21 (4.6%)
Thyroid	30 (6.6%)
Testicular	17 (3.7%)
Uterine	14 (3.1%)
Ovarian	19 (4.2%)
Sarcoma	6 (1.3%)
Kidney	5 (1.1%)
Bladder	4 (0.9%)
Other	21 (4.6%)
Country of residence	
USA	162 (35.5%)
UK	108 (23.7%)
South Africa	106 (23.2%)
Others	80 (17.5%)
Treatments received	
Chemotherapy	307 (67.3%)
Radiation	239 (53.4%)
Hormone treatment	123 (27.0%)
Targeted Therapies	63 (13.8%)
Surgery	108 (23.7%)
Immunotherapy	71 (15.6%)
Other	15 (3.3%)
Years since diagnosis	8.9 (7.6)

Rounding and multiple treatment modalities may result in percentages not equalling 100%

### Bivariate correlations

Bivariate correlations between all variables were conducted to identify the clinical, demographic, cognitive, and psychological factors that are associated with CRCI-related unmet needs. The associations are presented in Table 2, and coloured according to their strength and direction of association. The PROMIS-COG and all three DASS-21 subscales were significantly positively associated with the MASCC COG-IMPACT unmet needs overall score and all subscales with medium strengths (PROMIS-COG: *r* = 0.39 to 0.59, *p* < 0.001; DASS-21: *r* = 0.36 to 0.58, *p* < 0.001). The number of days since initial cancer diagnosis was also significantly negatively associated with the MASCC COG-IMPACT overall unmet needs score and all subscales, with small strengths (*r* =  − 0.11 to − 0.20; *p* < 0.001 to *p* = 0.02).

**Table 2:**
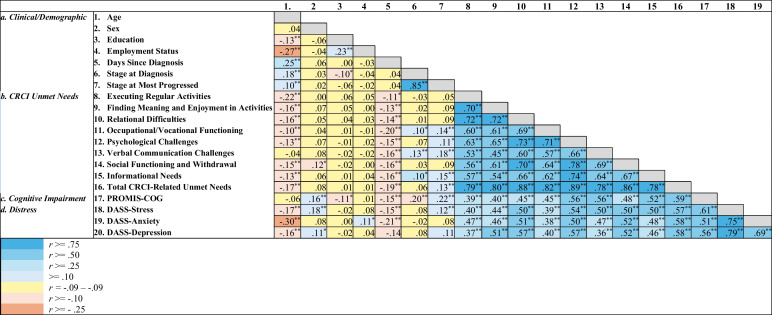
Bivariate correlations between all variables

*Significant at the 0.05 level (two-tailed). **Significant at the 0.01 level (two-tailed). Sex = 1 (male), 2 (female). Employment status = 1 (no), 2 (yes).

Age was significantly negatively associated with the MASCC COG-IMPACT unmet needs overall score and all of the MASCC COG-IMPACT unmet needs subscales, apart from verbal communication challenges, with a small strength (*r* =  − 0.10 to − 0.22; *p* < 0.001 to *p* = 0.03). Cancer stage at its most progressed was significantly positively associated with the MASCC COG-IMPACT overall unmet needs score as well as occupational/vocational functioning, psychological challenges, verbal communication challenges, and informational needs subscales, with small strengths (*r* = 0.11 to 0.18; *p* < 0.001 to *p* = 0.03).

Cancer stage at initial diagnosis was significantly positively associated with only three MASCC COG-IMPACT subscales, occupational/vocational functioning, verbal communication challenges, and informational needs, with small strengths (*r* = 0.10 to 0.13; *p* < 0.001 to *p* = 0.04). Sex was only significantly associated with the MASCC COG-IMPACT social functioning and withdrawal unmet needs subscale, with a small strength (*r* = 0.12, *p* = 0.01), with females experiencing greater unmet needs. Finally, level of education and employment status were not significantly associated with any MASCC COG-IMPACT subscale.

### Feed-forward multiple regression analyses

Each feed-forward multiple regression analysis to establish the most pertinent clinical, demographic, cognitive, and/or psychological variables that account for CRCI-related unmet needs is presented in Table 3, and described below according to each MASCC COG-IMPACT subscale and the overall score.

**Table 3:** Feed-forward multiple regressions

Model	Unstandardised coefficients		Standardised Coefficients	*t*	Sig	Correlations		
	B	Std. Error	Beta			Zero-order	Partial	Part
*Executing regular activities*								
Anxiety	0.019	0.003	0.323	6.51	< 0.001	0.466	0.293	0.264
Cognitive impairment	0.015	0.003	0.216	4.56	< 0.001	0.387	0.210	0.185
Age	− 0.004	0.002	− 0.112	− 2.61	0.009	− 0.222	− 0.122	− 0.106
*Finding meeting and enjoyment in activities*								
Depression	0.020	0.004	0.310	5.34	< 0.001	0.507	0.244	0.211
Cognitive impairment	0.013	0.004	0.147	3.02	0.003	0.404	0.141	0.120
Anxiety	0.012	0.004	0.166	2.96	0.003	0.456	0.138	0.117
*Relational difficulties*								
Anxiety	0.018	0.004	0.267	4.89	< 0.001	0.509	0.224	0.189
Cognitive impairment	0.016	0.004	0.206	4.34	< 0.001	0.452	0.200	0.167
Depression	0.012	0.003	0.198	3.49	< 0.001	0.498	0.162	0.135
*Occupational/vocational functioning*								
Cognitive impairment	0.022	0.004	0.299	5.96	< 0.001	0.449	0.270	0.243
Depression	0.009	0.003	0.154	2.56	0.011	0.407	0.119	0.105
Anxiety	0.008	0.004	0.126	2.17	0.030	0.384	0.102	0.089
*Psychological challenges*								
Depression	0.021	0.004	0.288	5.46	< 0.001	0.566	0.249	0.196
Cognitive impairment	0.031	0.004	0.329	7.44	< 0.001	0.559	0.330	0.267
Anxiety	0.011	0.004	0.137	2.69	0.007	0.504	0.125	0.097
*Verbal communication challenges*								
Cognitive impairment	0.033	0.004	0.401	8.45	< 0.001	0.564	0.370	0.316
Anxiety	0.012	0.004	0.173	3.04	0.003	0.469	0.141	0.113
Stress	0.009	0.004	0.123	1.99	0.047	0.498	0.093	0.074
*Social functioning and withdrawal*								
Anxiety	0.020	0.004	0.254	4.73	< 0.001	0.519	0.217	0.179
Cognitive impairment	0.021	0.004	0.226	4.86	< 0.001	0.476	0.223	0.184
Depression	0.015	0.004	0.216	3.88	< 0.001	0.518	0.180	0.147
*Informational needs*								
Cognitive impairment	0.025	0.004	0.321	6.64	< 0.001	0.519	0.298	0.253
Anxiety	0.014	0.004	0.206	3.56	< 0.001	0.483	0.165	0.136
Stress	0.010	0.004	0.152	2.40	0.017	0.503	0.112	0.092
*Overall CRCI-related unmet needs*								
Cognitive impairment	0.021	0.003	0.336	8.00	< 0.001	0.588	0.352	0.273
Anxiety	0.014	0.003	0.258	5.35	< 0.001	0.580	0.244	0.182
Depression	0.010	0.002	0.217	4.33	< 0.001	0.583	0.200	0.148

#### Executing regular activities

The final model accounting for executing regular activities CRCI-related unmet needs included the DASS-Anxiety, the PROMIS-COG, and age. In the final model, which accounted for a significant 25.9% of the variance in executing regular activities CRCI-related unmet needs (*R*^2^ = 0.259, *F*_(3, 487)_ = 52.63, *p* < 0.001), greater anxiety, greater cognitive impairment, and lower age, were associated with greater unmet needs. Anxiety accounted for the most unique variance in executing regular activities CRCI-related unmet needs (6.49%), followed by cognitive impairment (3.42%), and age (1.11%).

#### Finding meaning and enjoyment in activities

The final model accounting for finding meaning and enjoyment in activities CRCI-related unmet needs included DASS-Depression, the PROMIS-COG, and DASS-Anxiety. In the final model, which accounted for a significant 29.3% of the variance in finding meaning and enjoyment in activities CRCI-related unmet needs (*R*^2^ = 0.293, *F*_(3, 487)_ = 62.35, *p* < 0.001), greater depression, greater cognitive impairment, and greater anxiety were associated with greater unmet needs. Depression accounted for the most unique variance in finding meaning and enjoyment activities CRCI-related unmet needs (4.45%), followed by cognitive impairment (1.44%), and anxiety (1.37%).

#### Relational difficulties

The final model accounting for relational difficulties CRCI-related unmet needs included the DASS-Anxiety, the PROMIS-COG, and DASS-Depression. In the final model, which accounted for a significant 32.7% of the variance in relational difficulties CRCI-related unmet needs (*R*^2^ = 0.327, *F*_(3, 487)_ = 73.37, *p* < 0.001), greater anxiety, greater cognitive impairment, and greater depression, were associated with greater unmet needs. Anxiety accounted for the most unique variance in relational difficulties CRCI-related unmet needs (3.57%), followed by cognitive impairment (2.79%), and depression (1.82%).

#### Occupational/vocational functioning

The final model accounting for occupational/vocational functioning CRCI-related unmet needs included the PROMIS-COG, DASS-Depression, and DASS-Anxiety. In the final model, which accounted for a significant 24.5% of the variance in occupational/vocational functioning CRCI-related unmet needs (*R*^2^ = 0.245, *F*_(3, 487)_ = 48.96, *p* < 0.001), greater cognitive impairment, greater depression, and greater anxiety were associated with greater unmet needs. Cognitive impairment accounted for the most unique variance in occupational/vocational functioning CRCI-related unmet needs (5.90%), followed by depression (1.10%), and anxiety (0.79%).

#### Psychological challenges

The final model accounting for psychological challenges CRCI-related unmet needs included the DASS-Depression, PROMIS-COG, and DASS-Anxiety. In the final model, which accounted for a significant 41.6% of the variance in psychological challenges CRCI-related unmet needs (*R*^2^ = 0.416, *F*_(3, 487)_ = 107.35, *p* < 0.001), greater depression, greater cognitive impairment, and greater anxiety were associated with greater unmet needs. Cognitive impairment accounted for the most unique variance in psychological challenges CRCI-related unmet needs (7.13%), followed by depression (3.84%), and anxiety (0.94%).

#### Verbal communication challenges

The final model accounting for verbal communication challenges CRCI-related unmet needs included the PROMIS-COG, DASS-Anxiety, and DASS-Stress. In the final model, which accounted for a significant 36.9% of the variance in verbal communication challenges CRCI-related unmet needs (*R*^2^ = 0.369, *F*_(3, 487)_ = 87.94, *p* < 0.001), greater cognitive impairment, greater anxiety, and greater stress were associated with greater unmet needs. Cognitive impairment accounted for the most unique variance in verbal communication challenges CRCI-related unmet needs (10.00%), followed by anxiety (1.28%), and stress (0.55%).

#### Social functioning and withdrawal

The final model accounting for social functioning and withdrawal CRCI-related unmet needs included DASS-Anxiety, the PROMIS-COG, and DASS-Depression. In the final model, which accounted for a significant 35.1% of the variance in social functioning and withdrawal CRCI-related unmet needs (*R*^2^ = 0.351, *F*_(3, 487)_ = 81.56, *p* < 0.001). Greater anxiety, greater cognitive impairment, and greater depression were associated with greater unmet needs. Cognitive impairment accounted for the most unique variance in social functioning and withdrawal CRCI-related unmet needs (3.40%), followed by anxiety (3.20%), and depression (2.16%).

#### Informational needs

The final model accounting for informational CRCI-related unmet needs included the PROMIS-COG, DASS-Anxiety, and DASS-Stress. In the final model, which accounted for a significant 34.3% of the variance in informational CRCI-related unmet needs (*R*^2^ = 0.343, *F*_(3, 487)_ = 78.57, *p* < 0.001), greater cognitive impairment, greater anxiety, and greater stress, were associated with greater unmet needs. Cognitive impairment accounted for the most unique variance in informational CRCI-related unmet needs (6.40%), followed by anxiety (1.85%), and stress (0.85%).

#### Overall CRCI-related unmet needs

The final model for overall CRCI-related unmet needs included DASS-Anxiety, PROMIS-COG, DASS-Anxiety, and DASS-Depression. In the final model, which accounted for a significant 47.4% of the variance in overall CRCI-related unmet needs (*R*^2^ = 0.474, *F*_(3, 487)_ = 114.81, *p* < 0.001), greater cognitive impairment, greater anxiety, and greater depression were associated with greater unmet needs. Cognitive impairment accounted for the most unique variance in overall CRCI-related unmet needs (7.45%), followed by anxiety (3.31%), and depression (2.19%).

## Discussion

The aims of this study were to identify the clinical, demographic, cognitive, and psychological factors *associated* with CRCI-related unmet needs, and to establish the most pertinent clinical, demographic, cognitive, and psychological variables that *account* for CRCI-related unmet needs.

Cognitive impairment severity, depression, anxiety, stress, and days since initial diagnosis were significantly associated with all domains of CRCI-related unmet needs. The association between the severity of cognitive impairment and CRCI-related unmet needs was expected, as greater cognitive challenges are associated with poorer outcomes across quality-of-life domains in cancer survivorship [3, 7, 9, 39], and therefore cancer survivors are likely to experience CRCI-related unmet needs across these domains. Similarly, psychological distress (i.e. depression, anxiety, and stress) is common in cancer survivorship and is associated with poor outcomes across domains such as occupational and vocational functioning, social functioning, and activities of daily living [23, 24, 40]. Further, it has been proposed that there is a functional bidirectional relationship between psychological distress and CRCI (i.e. both distress and CRCI precipitating and/or perpetuating one another) [3, 41]. While the body of literature establishing the existence of a functional relationship between distress and CRCI is still growing [3, 41], there is strong evidence in non-cancer populations that psychological distress and cognition are functionally related [29, 42–45]. Therefore, taking the larger body of literature into account, it is likely that CRCI and mental health challenges bidirectionally contribute toward one another, and mental health challenges exacerbate CRCI-related unmet needs across domains. In line with the literature on general unmet needs in cancer survivorship [20–22], it was also observed that additional time since diagnosis was significantly associated with all domains of CRCI-related unmet needs. Additional time since initial diagnosis may be associated with fewer CRCI unmet needs due to extended clinical and practical rehabilitation and management strategies increasingly minimising the severity of CRCI and CRCI impact over time.

The regression models found that cognitive impairment severity, as well as at least one domain of psychological distress, were pertinent factors accounting for unique variance across each domain of CRCI-related unmet needs. This suggests the possibility that CRCI and psychological distress may not only be functionally related [3], but psychological distress may contribute toward CRCI-related unmet needs independently from cognitive impairment severity, although future longitudinal research is required. Psychological distress is known to impact self-efficacy, self-perception, expectation-perception, and motivation [25, 26]. Therefore, psychological distress may be associated with CRCI-related unmet needs due to its impact on both the perception of self and the perception of the expectation of others with regard to changes in their cognitive capabilities. In line with this proposition, qualitative research has noted CRCI can result in changes to self-identity, self-efficacy, and the perception of others’ expectations [8, 9, 15].

These findings suggest that while a large number of clinical, demographic, cognitive, and psychological variables are associated with CRCI-related unmet needs, only a subset of variables are required to significantly account for CRCI-related unmet needs across the eight domains to the greatest predictive power. In particular, cognitive and psychological distress measures provided more utility in accounting for CRCI-related unmet needs than any other demographic or clinical variable tested. The pertinence of key factors in accounting for CRCI-related unmet needs outlined in this study may inform supportive care practice and research. For example, in the clinical setting, screening for CRCI-related unmet needs might be prioritised for cancer survivors who present with subjective cognitive impairment as well as psychological distress. In the research setting, the identified key factors in accounting for CRCI-related unmet needs may be further explored in longitudinal work to examine the potential for functional associations.

### Strengths, limitations, and directs for future research

This study had several key strengths. The study utilised a large sample of cancer survivors with diverse and global representations across clinical and demographic characteristics including age, sex, ethnicity, country, cancer type, cancer stage, and treatments received. This study also used rigorous methods and statistical techniques used across fields [e.g. 46, thus facilitating strong conclusions. This study, however, also had some key limitations. This sample is limited to only cancer survivors who have completed curative-intent treatment and are currently free of cancer. Future research should consider cancer survivors from other sectors of the cancer care continuum, including those currently undergoing treatment with curative intent as well as those with advanced cancer or metastatic disease. The sample consists of cancer survivors from a large number of tumour types. While this facilitates the exploration of this population broadly, it also limits the potential to understand associations within specific tumour types. Future research should consider the nuances assessment and comparisons of and between specific tumour types. All data-points, including clinical data, were self-reported which increases the potential for error in reporting. Future research may utilise medical records. Further, there we did not collect a complete indication of the cancer survivors’ clinical descriptors, including no collection of data points which as body mass index, blood pressure, and comorbidities). There is potential for some of these indicators to moderate or mediate relationships between the included variables and CRCI and CRCI-related unmet needs [47], and therefore future research should collect a broader collection of clinical descriptors. In line with the body of literature within survivorship, the majority of participants were female. Future research may purposefully sample additional male participants. We did not assess CRCI through objective measures, therefore we cannot determine with the participants would reach an established threshold for objectively measured CRCI and objective CRCI was not able to be included as a predictor variable within this research. Future research should explore objectively measured and determined CRCI within the exploration of CRCI-related unmet needs. Cancer types were not able to be entered in the regression models as adding nominal variables with more than two categories are inappropriate in these analyses. Future research should parametric techniques to examine CRCI-unmet needs in relation to cancer types. We utilised a cross-sectional approach, which is useful in developing this initial body of literature in this area, but is unable to be used to explore questions of order of occurrence and causality. Future research should develop and utilise longitudinal data using the MASCC COG-IMPACT. Lastly, we used a feed-forward regression approach. This approach is useful to establish the smallest number of variables that account for the maximum proportion of variance in CRCI-related unmet needs. The establishment of a small collection of variables with large predictive utility is particularly useful to inform the choice of screening and supportive care targets, among many potential targets. However, the approach does have limitations. It is data-driven and a-theoretical, not allowing for the inclusion of particular variables based on theory [48, 49]. Future research may utilise other techniques, such as machine learning.

## Conclusion

Cognitive impairment severity and psychological distress are the most pertinent factors in accounting for CRCI-related unmet needs across domains. Other clinical and demographic variables, such as time since diagnosis, cancer stage, and sex, while associated with CRCI-related unmet needs, did not provide predictive utility beyond cognitive impairment severity and psychological distress measures. These results provide key information that may inform the choice of screening and supportive care targets to improve the quality of life of people experiencing CRCI.

## Data Availability

Data may be made available upon request of the corresponding author and to the satisfaction of the HREC.

## References

[1] Moreno AM, Hamilton RA, Currier MB. Cancer-related cognitive impairment: diagnosis, pathogenesis, and management. In: Breast Cancer and Gynecologic Cancer Rehabilitation. Elsevier; 2021. p. 211–23.

[2] Bray, V.J., H.M. Dhillon, and J.L. Vardy. Cancer-related cognitive impairment in adult cancer survivors: a review of the literature. 2017.

[3] Haywood D, et al. Cancer-related cognitive impairment as a key contributor to psychopathology in cancer survivors: implications for prevention, treatment and supportive care. Support Care Cancer. 2024;32(7):1–5. 10.1007/s00520-024-08696-9 PMC11219369 38954104

[4] Haywood D, Henneghan AM, Chan A, Chan RJ, Dhillon HM, Lustberg MB, et al. The effect of non-pharmacological interventions on cognitive function in cancer: an overview of systematic reviews. Support Care Cancer. 2025;33(2):151. 10.1007/s00520-025-09212-3 PMC11794363 39904905

[5] Binarelli G, et al. Cancer-related cognitive impairment: current perspectives on the management of cognitive changes following cancer treatment. Expert Rev Neurother. 2023;23(3):249–68. 36951414 10.1080/14737175.2023.2187288

[6] Haywood DBaughman FD, Dauer E, Haywood J, Rossell S, Hart NH. It’s about time: mitigating cancer-related cognitive impairments through findings from computational models of the Wisconsin card sorting task. BMC Cancer. 2024;24(1):798. 10.1186/s12885-024-12545-7 PMC11223404 38965483

[7] Henneghan AM, Van Dyk KM, Haywood D, Patel M, Franco-Rocha OY, Bang S, et al. Characterizing cancer-related cognitive impairments and impact on quality of life in women with metastatic breast cancer. Breast Cancer Res Treat. 2024;13:1–14. 10.1007/s10549-024-07479-4 PMC12308896 39269553

[8] Haywood D, et al. Oncology healthcare professionals’ perceptions and experiences of ‘chemobrain’in cancer survivors and persons undergoing cancer treatment. Gen Hosp Psychiatry. 2023; S0163-8343. 10.1016/j.genhosppsych.2023.06.017 37407422

[9] Haywood D, et al. “Is my brain ever going to work fully again?”: challenges and needs of cancer survivors with persistent cancer-related cognitive impairment. Cancers. 2023;15(22):5331. 38001592 10.3390/cancers15225331 PMC10669848

[10] Klaver KM, et al. Neuropsychological test performance and self-reported cognitive functioning associated with work-related outcomes in occupationally active cancer survivors with cognitive complaints. J Cancer Surviv. 2024;18(2):412–24. 35776235 10.1007/s11764-022-01223-x

[11] Von Ah D, et al. Association of cognitive impairment and breast cancer survivorship on quality of life in younger breast cancer survivors. J Cancer Surviv. 2022;16(4):812–22. 34173970 10.1007/s11764-021-01075-x PMC9300496

[12] Haywood D, et al. Protocol for the development and initial validation of the COG-IMPACT tool: a purpose-built unmet needs assessment for cancer-related cognitive impairment. Methods Protocols. 2024;7(4). 10.3390/mps7040054 PMC11270296 39051268

[13] Hart NH, et al. World health organization package of interventions for rehabilitation for cancer: a MASCC-endorsed resource for global action to address unmet rehabilitation needs of people affected by cancer. Support Care Cancer. 2024;32(7):417. 38847912 10.1007/s00520-024-08569-1

[14] Fan R, et al. Unmet supportive care needs of breast cancer survivors: a systematic scoping review. BMC Cancer. 2023;23(1):587. 37365504 10.1186/s12885-023-11087-8 PMC10294377

[15] Henderson FME, Cross AJ, Baraniak AR. ‘A new normal with chemobrain’: experiences of the impact of chemotherapy-related cognitive deficits in long-term breast cancer survivors. Health Psychol Open. 2019;6(1):2055102919832234. 30873289 10.1177/2055102919832234 PMC6405778

[16] Haywood D, Chan A, Chan RJ, Baughman FD, Dauer E, Dhillon HM, et al. The MASCC COG-IMPACT: an unmet needs assessment for cancer-related cognitive impairment impact developed by the multinational association of supportive care in cancer. Support Care Cancer. 2025;33(2):120. 10.1007/s00520-025-09149-7 PMC11761510 39853439

[17] Fan R, et al. Unmet supportive care needs of breast cancer survivors: a systematic scoping review. BMC Cancer. 2023;23(1):1–24. 37365504 10.1186/s12885-023-11087-8 PMC10294377

[18] Hart NH, et al. Unmet supportive care needs of people with advanced cancer and their caregivers: a systematic scoping review. Crit Rev Oncol Hematol. 2022;176:103728. 35662585 10.1016/j.critrevonc.2022.103728

[19] Herzog AR, Ofstedal MB, Wheeler LM. Social engagement and its relationship to health. Clin Geriatr Med. 2002;18(3):593–609. 12424874 10.1016/s0749-0690(02)00025-3

[20] Millar B, Patterson P, Desille N. Emerging adulthood and cancer: how unmet needs vary with time-since-treatment. Palliat Support Care. 2010;8(2):151–8. 20307366 10.1017/S1478951509990903

[21] Miroševič Š, et al. Prevalence and factors associated with unmet needs in post-treatment cancer survivors: a systematic review. Eur J Cancer Care. 2019;28(3):e13060. 10.1111/ecc.13060 31008544

[22] Willems RA, et al. Cancer survivors in the first year after treatment: the prevalence and correlates of unmet needs in different domains. Psychooncology. 2016;25(1):51–7. 26110652 10.1002/pon.3870

[23] Haywood D, Kotov R, Krueger RF, Wright AG, Forbes MK, Dauer E, et al. Is it time to discard the diagnostic and statistical manual of mental disorders (DSM) in psycho-oncology? Cancer Lett. 2024;216818–216818. 10.1016/j.canlet.2024.216818 38554804

[24] Haywood, D., et al., Reconceptualizing mental health in cancer survivorship. Trends in Cancer, 2024. 10.1016/j.trecan.2024.05.008 38890021

[25] LeMoult J, Gotlib IH. Depression: a cognitive perspective. Clin Psychol Rev. 2019;69:51–66. 29961601 10.1016/j.cpr.2018.06.008 PMC11884012

[26] Hames JL, Hagan CR, Joiner TE. Interpersonal processes in depression. Annu Rev Clin Psychol. 2013;9(1):355–77. 23297787 10.1146/annurev-clinpsy-050212-185553

[27] Palan S, Schitter C. Prolific. ac—a subject pool for online experiments. J Behav Exp Finance. 2018;17:22–7.

[28] Haywood D, et al. Do dimensional measures of mental health symptoms predict level of alcohol involvement in the general population? Subst Use Misuse. 2023;58(5):629–36. 36790047 10.1080/10826084.2023.2177962

[29] Haywood D, Baughman FD, Mullan BA, Heslop KR. What accounts for the factors of psychopathology? An investigation of the neurocognitive correlates of internalising, externalising, and the p-factor. Brain Sci. 2022;12(4):421. 10.3390/brainsci12040421 PMC9030002 35447951

[30] Haywood D, et al. Neurocognitive artificial neural network models are superior to linear models at accounting for dimensional psychopathology. Brain Sci. 2022;12(8):1060. 36009123 10.3390/brainsci12081060 PMC9405994

[31] Uittenhove K, Jeanneret S, Vergauwe E. From lab-based to web-based behavioural research: Who you test is more important than how you test. J Cogn. 2022. 10.31234/osf.io/uy4kb. 10.5334/joc.259 PMC9854315 36721797

[32] Riley WT, Pilkonis P, Cella D. Application of the National Institutes of Health patient-reported outcomes measurement information system (PROMIS®) to mental health research. J Ment Health Policy Econ. 2011;14(4):201. 22345362 PMC3705221

[33] Henry JD, Crawford JR. The short-form version of the depression anxiety stress scales (DASS-21): construct validity and normative data in a large non-clinical sample. Br J Clin Psychol. 2005;44(2):227–39. 16004657 10.1348/014466505X29657

[34] Haywood D, et al. The MASCC COG-IMPACT: an unmet needs assessment for cancer-related cognitive impairment impact developed by the multinational association of supportive care in cancer. Support Care Cancer. 2025;33(2):120. 39853439 10.1007/s00520-025-09149-7 PMC11761510

[35] Henneghan AM, et al. Validating the PROMIS cognitive function short form in cancer survivors. Breast Cancer Res Treat. 2023;201(1):139–45. 37330430 10.1007/s10549-023-06968-2 PMC10729147

[36] Henneghan AM, et al. Measuring self-reported cancer-related cognitive impairment: recommendations from the cancer neuroscience initiative working group. JNCI J Nat Cancer Inst. 2021;113(12):1625–33. 33638633 10.1093/jnci/djab027 PMC8849125

[37] Dancey CP, Reidy J. Statistics without maths for psychology. 5th ed. Prentice Hall: Pearson Education Limited; 2007. pp. 461–472.

[38] Sedgwick P. Multiple significance tests: the Bonferroni correction. Bmj. 2012;25:344–e509. 10.1136/bmj.e509.

[39] Bhatt VR, et al. Longitudinal changes in cognitive and physical function and health-related quality of life in older adults with acute myeloid leukemia. J Geriatr Oncol. 2024;15(1):101676. 38000343 10.1016/j.jgo.2023.101676 PMC11101205

[40] Joshy G, et al. Disability, psychological distress and quality of life in relation to cancer diagnosis and cancer type: population-based Australian study of 22,505 cancer survivors and 244,000 people without cancer. BMC Med. 2020;18:1–15. 33256726 10.1186/s12916-020-01830-4 PMC7708114

[41] Vega JN, et al. Subjective cognition and mood in persistent chemotherapy-related cognitive impairment. J Cancer Surviv. 2022;16(3):614–23. 33973154 10.1007/s11764-021-01055-1 PMC9390086

[42] Haywood D, et al. Going “Up” to move forward: S-1 bifactor models and the study of neurocognitive abilities in psychopathology. Int J Environ Res Public Health. 2021;18(14):7413. 34299862 10.3390/ijerph18147413 PMC8307957

[43] Haywood D, et al. Psychopathology and neurocognition in the era of the p-factor: the current landscape and the road forward. Psychiatry Int. 2021;2(3):233–49.

[44] Haywood D, Baughman FD. Multidimensionality in executive function profiles in schizophrenia: a computational approach using the Wisconsin card sorting task. Comput Brain Behav. 2021;4(4):381–394.

[45] Snyder HR. Major depressive disorder is associated with broad impairments on neuropsychological measures of executive function: a meta-analysis and review. Psychol Bull. 2013;139(1):81. 22642228 10.1037/a0028727 PMC3436964

[46] Haywood D, et al. What accounts for turnover intention in the Australian public mental health workforce? Int J Ment Health Nurs. 2024;33(2):359–68. 37795874 10.1111/inm.13233

[47] Mayo SJ, et al. Cancer-related cognitive impairment in patients with non-central nervous system malignancies: an overview for oncology providers from the MASCC Neurological Complications Study Group. Support Care Cancer. 2021;29:2821–40. 33231809 10.1007/s00520-020-05860-9

[48] Flom PL, Cassell DL. Stopping stepwise: why stepwise and similar selection methods are bad, and what you should use. In: NorthEast SAS Users Group Inc 20th Annual Conference; 2007. pp. 11–14.

[49] Harrell FE. Regression modeling strategies: with applications to linear models, logistic regression, and survival analysis. New York: Springer; 2001.

